# Who Is (Not) Engaging With a Digital Parenting Intervention in Tanzania? Analysis of Predictors Using Data From a Factorial Randomized Trial

**DOI:** 10.2196/86129

**Published:** 2026-09-22

**Authors:** Roselinde Janowski, Lucie D Cluver, Joyce Wamoyi, G J Melendez-Torres, Jamie M Lachman, David Stern, Lauren-Jayne van Niekerk, Yulia Shenderovich

**Affiliations:** 1 Department of Social Policy and Intervention, University of Oxford, Barnett House, 32 -37 Wellington Square, Oxford, OX1 2ER, United Kingdom, 44 01865 270325; 2 Department of Psychiatry and Mental Health, University of Cape Town, Cape Town, South Africa; 3 Mwanza Research Centre, National Institute for Medical Research, Mwanza, United Republic of Tanzania; 4 Department of Public Health and Sport Sciences, Faculty of Health and Life Sciences, University of Exeter, Exeter, United Kingdom; 5 Parenting for Lifelong Health, Oxford, United Kingdom; 6 Centre for Social Science Research, University of Cape Town, Cape Town, South Africa; 7 IDEMS International Community Interest Company, Reading, United Kingdom; 8 Centre for Development, Evaluation, Complexity, and Implementation in Public Health Improvement (DECIPHer), School of Social Sciences, Cardiff University, Cardiff, United Kingdom; 9 Wolfson Centre for Young People’s Mental Health, Cardiff University, Cardiff, United Kingdom

**Keywords:** digital health, mobile phone, parenting, violence against children, predictors, engagement, low- and middle-income countries

## Abstract

**Background:**

Digital interventions offer a promising avenue for scalable parenting support in low- and middle-income countries, where in-person coverage remains limited. However, engagement disparities may reduce their potential for population-level impact.

**Objective:**

This study examined caregiver-level predictors of engagement with ParentApp, a smartphone-based adaptation of the Parenting for Lifelong Health (PLH) for Teens program for caregivers of adolescents aged 10 to 17 years.

**Methods:**

This study was nested within a cluster-randomized factorial trial of ParentApp in Mwanza, Tanzania, implemented according to the optimization phase of the Multiphase Optimization Strategy. Clusters (n=16), comprising 614 caregivers (n=205, 33.4% men), were randomized to 3 components: guidance (guided vs self-guided), app design (unstructured vs structured), and digital support (enhanced vs basic). Digitally tracked app engagement outcomes included the number of modules completed, the first module not started, and the first module not completed. Baseline candidate predictors included demographic, socioeconomic, behavioral, and psychological factors assessed via an app-embedded questionnaire. Poisson generalized linear mixed-effects models with random effects for clusters assessed associations between predictors and each outcome. Interaction terms were specified to test whether associations differed by caregiver gender.

**Results:**

Among 614 caregivers, 596 (97.1%) completed the first module; however, engagement declined rapidly thereafter, with only 281 (45.8%) completing module 2 and 49 (7.9%) completing all 12 modules. Overall, mean content completion was 35.6% (SD 33.1%) of the program. Women completed approximately 15% more modules than men (incidence rate ratio 1.15, 95% CI 1.05-1.27, *P*=.003), despite reporting higher levels of financial stress, food insecurity, child maltreatment, and depressive symptoms at baseline (all *P*≤.02; Cohen *d*=0.19‐0.37). No other caregiver characteristics were significantly associated with engagement.

**Conclusions:**

This is the first known study to investigate caregiver-level predictors of engagement with a digital parenting intervention in a low- and middle-income country. Engagement with ParentApp was broadly consistent across caregiver subgroups, with the exception that women completed significantly more modules than men. To equitably engage men and fathers, future studies should pair father-inclusive, identity-affirming content with implementation strategies that reduce social and economic barriers to participation.

## Introduction

Parenting interventions are central to global efforts to prevent violence against children and promote family well-being [1,2]. However, access to evidence-based parenting support remains limited in many low- and middle-income countries (LMICs). Workforce shortages and the high costs of in-person delivery restrict service availability, while travel, time, and competing work and caregiving responsibilities make it difficult for many families to participate [3,4]. Promisingly, there is growing evidence that digital parenting interventions can improve parenting and child outcomes, including among hard-to-reach families in LMICs [5,6]. However, program effects are often small and user engagement is low [7], warranting investigation into how these interventions can be designed and implemented to sustain engagement and achieve population-level impact.

Engagement is a key mechanism for behavioral change [8]. Research on engagement with digital interventions has largely been conducted within behavioral science, which typically defines engagement as the extent of program use (eg, amount, frequency, duration, and depth), although its operationalization varies widely across studies [9]. While engagement challenges are not unique to digital delivery, they may be amplified by structural inequalities in digital access and use, particularly in LMICs.

Families living in poverty or rural areas often rely on lower-performing devices, have limited internet connectivity, and face digital literacy challenges [10]. In Tanzania, for example, 82% of the population owns a basic mobile phone, compared with just 35% who own a smartphone [11]. Gender disparities in digital access further compound these barriers. Across LMICs, women are 17% less likely to own a mobile phone and 21% less likely to use the internet than men [12]. These digital divides risk excluding already marginalized families and may deepen health and social inequities if unaddressed. Identifying which caregivers are most likely to engage, or disengage, is therefore essential for developing equitable and effective digital parenting interventions.

Existing evidence on caregiver engagement with digital parenting programs remains limited and inconsistent, with relatively few studies conducted in LMICs [7]. Gender disparities are well documented in in-person programs, where fathers engage at markedly lower rates than mothers [13,14]. In a South African trial of the in-person Parenting for Lifelong Health (PLH) Teens program, fathers represented only 3% of participants and were significantly less likely to attend sessions than mothers [15]. Encouragingly, early evidence from high-income countries (HICs) suggests mothers and fathers engage at similar rates in digitally delivered parenting programs [16,17]. Whether these findings extend to LMICs remains unknown.

Beyond gender, evidence on caregiver-level predictors remains limited. Sociodemographic factors appear to be the most extensively studied in digital parenting interventions, with lower education and income consistently linked to reduced engagement and increased attrition in HICs [17-21]. Similar associations have been reported in in-person parenting programs in HICs [22]. By contrast, findings from a few in-person programs in LMICs suggest that socioeconomic disadvantage does not reliably predict lower engagement [15]. Evidence on behavioral and psychological predictors is comparatively limited and mixed, with positive parenting practices [21,23] and caregiver mental health [16,20,21,24] both associated with higher and lower engagement in digital parenting programs. To our knowledge, no studies of digital parenting interventions have examined these associations in LMICs.

The current study therefore examines caregiver-level predictors of engagement in an early version of ParentApp, a digital parenting intervention developed for caregivers of adolescents in LMICs. Using data from a cluster-randomized factorial trial in Tanzania, implemented in accordance with the optimization phase of the Multiphase Optimization Strategy (MOST) [25], we test whether caregiver demographic, socioeconomic, behavioral, and psychological characteristics predict user engagement. We also explore whether these associations differ by caregiver gender. Findings are intended to inform targeted and inclusive strategies to enhance engagement in ParentApp and, more broadly, support the equitable scale-up of digital parenting interventions in LMICs.

## Methods

### Study Design

This study was nested within a 2 × 2 × 2 cluster-randomized full factorial experiment implemented across 16 low-income urban and periurban communities in Mwanza, Tanzania [26]. Each community (cluster) was randomly assigned to one of 8 experimental conditions, comprising all possible combinations of 3 components: guidance (guided vs self-guided), app design (structured vs unstructured), and digital support (enhanced vs basic). The trial aimed to optimize user engagement with ParentApp, which was developed in partnership with the United Nations Children’s Fund, the World Health Organization, and the Tanzanian national government. The trial was preregistered on the Pan-African Clinical Trial Registry (PACTR202210657553944) on October 11, 2022. Further information about the trial design and findings can be found in the study protocol and related trial publications [26-28].

### Participants

Eligible participants were required to be at least 18 years old, be caring for an adolescent aged 10 to 17 years, reside in the same household as the adolescent for at least 4 nights per week during the previous month, and have regular access to an Android smartphone (OS 5.1.1 or higher). To address the underrepresentation of fathers in parenting interventions, the study aimed to enroll at least 20% men. Participants were recruited on a rolling basis between October 24, 2022 and December 1, 2022, through local community leaders, who were predominantly men. Community leaders introduced and endorsed the study during community meetings, helping to build trust and acceptability among families.

### Interventions

#### ParentApp

ParentApp is designed to promote positive parenting, prevent violence against adolescents, and reduce risks of sexual violence victimization. It is a smartphone-based adaptation of the in-person PLH Teens program, originally developed and evaluated in South Africa [29] and subsequently implemented across multiple LMICs, including Tanzania [30]. ParentApp was developed to expand reach and reduce costs and logistical barriers of in-person delivery. To support use in settings with limited or intermittent internet access, it operates offline after the initial download.

Development and testing followed a multiphase process guided by the MOST framework [25]. During the preparation phase, co-design, multicountry user testing [31], and pilot studies in South Africa and Tanzania demonstrated that the app was broadly acceptable. The preparation phase also identified opportunities to strengthen delivery through additional human support, greater flexibility in accessing content, culturally adapted imagery, and enhanced digital support for caregivers with lower smartphone literacy. These findings informed the selection of the 3 components evaluated in the optimization-phase factorial trial: guidance, app design, and digital support [26].

The intervention content tested in the factorial trial was organized into 12 modules, delivered via simple text, illustrations, and audio narration in Kiswahili. The introductory module focuses on parental self-care and stress reduction and includes a baseline questionnaire. Modules 2 to 11 introduce evidence-based strategies to support positive parenting and reduce harsh or abusive discipline, while also incorporating mindfulness-based exercises throughout. The final module provides a summary of key concepts and includes a postintervention assessment. Full details on module content, home practice activities, and user interface design are available in Janowski et al [26].

#### Experimental Components

##### Guidance

The guidance component compared self-guided use of ParentApp with facilitator-supported delivery. Following onboarding, participants in the self-guided condition used the app independently, whereas those in the guided condition used the app alongside a facilitator-moderated WhatsApp (Meta Platforms, Inc) group corresponding to their cluster allocation. Each group was cofacilitated by a mixed-gender facilitator pair to encourage engagement among both women and men. Following a standardized facilitation manual, facilitators posted weekly reminders and discussion prompts, responded to participants’ questions, moderated discussions, and hosted weekly 1-hour live chat sessions throughout the 12-week intervention period to support peer interaction and reflection.

##### App Design

The app design component compared a structured and an unstructured version of ParentApp. In the structured condition, modules were released sequentially over the 12-week intervention and retained the original abstract blue cartoon characters, which were designed without gender-specific characteristics and developed for cross-cultural implementation during earlier pilot studies. In the unstructured condition, all modules were available immediately following installation, allowing participants to progress through the content at their own pace, and featured culturally adapted, human-like illustrations depicting both women and men in caregiving roles.

##### Digital Support

All participants received basic digital support consisting of an embedded app tutorial and a group-based demonstration of key app features during onboarding. Clusters allocated to the enhanced digital support condition additionally received a 15‐ to 20-minute group-based training session designed to improve general smartphone literacy and users’ confidence in navigating their devices.

### Procedures

Cluster-level onboarding sessions were conducted in local schools and community centers by staff from Tanzania’s National Institute for Medical Research (NIMR) and trained facilitators from Investing in Children and Strengthening Their Societies, a local nongovernmental organization. Sessions included study information, eligibility screening, and informed consent, followed by app onboarding and baseline data collection. After obtaining informed consent, participants were divided into small groups (5‐10 caregivers) to facilitate hands-on support with downloading and installing ParentApp from the Google Play Store. Following installation, NIMR research assistants moved between participants to configure each app according to the cluster’s allocated intervention condition before guiding caregivers through the app’s core features, including navigation, activity completion, and available resources. Participants then completed the introductory module, which included the embedded baseline assessment. Clusters allocated to the enhanced digital support condition also participated in a brief group-based smartphone literacy session, while those allocated to the guided condition were added to facilitator-moderated WhatsApp groups.

### Measures

#### Engagement Metrics

Engagement was assessed using automatically recorded in-app usage data. Usage data, including app launches, content viewing patterns, and responses to in-app tasks, were uploaded to a Metabase cloud server when participants’ devices connected to the internet. Retention was calculated as the number of days between the first app launch and the final synchronization of app usage data with the cloud server. Additional usage data, including visit timestamps, session duration, and frequency of actions or pages viewed, were captured via Matomo Analytics (InnoCraft Ltd). These data were used to derive descriptive indicators and the primary and secondary outcomes.

The primary outcome was module completion, defined as the total number of modules completed (range 0‐12). Secondary outcomes included the first module not started and the first module not completed, which identified the earliest point in the intervention sequence at which participants either failed to initiate or failed to complete a module, respectively. These outcomes were selected to capture cumulative content engagement and early points of disengagement.

#### Candidate Predictors

##### Demographic and Socioeconomic Variables

Baseline data were collected via an in-app questionnaire embedded in the first module. Measures were translated and back-translated into Kiswahili. Candidate demographic predictors included caregiver gender (woman or man) and age (y). Additional demographics (eg, household composition) were collected but not analyzed here. Candidate socioeconomic predictors included financial stress and food insecurity, assessed using 2 items adapted from the Financial Self-Efficacy Scale [32]. Financial stress was assessed by asking, “How many times in the past month have you felt worried or anxious about money?” rated on a 0 to 8 or more frequency scale. Food insecurity was assessed by asking, “How many days in the past month did you run out of money to pay for food?” rated from 0 to 30 days. The 2 items were analyzed separately due to differences in response formats.

##### Behavioral and Psychological Variables

Positive parenting was assessed using 5 items on parental involvement and supervision from the Alabama Parenting Questionnaire [33]. Items were scored from 0 to 8 or more and summed to produce a total score, with higher scores indicating greater positive parenting. Harsh parenting was assessed using 4 items on physical and emotional abuse from the International Society for Prevention of Child Abuse and Neglect Screening Tools-Trial Version [34]. Items were scored from 0 to 8 or more and summed, with higher scores indicating greater maltreatment. Caregiver depression was measured using 3 items from the Center for Epidemiologic Studies Depression Scale [35]. Each item was scored from 0 to 7 and summed, with higher scores indicating greater depressive symptoms.

### Statistical Analysis

Analyses were conducted in R (version 4.5.0; R Foundation for Statistical Computing). Sample characteristics were summarized overall and by caregiver gender. Gender differences in age, financial stress, food insecurity, positive parenting, harsh parenting, and depressive symptoms were assessed using 2-tailed Welch independent-samples *t* tests, with Cohen *d* calculated to estimate effect sizes. Engagement across the 12 modules was visualized using Kaplan-Meier survival curves stratified by caregiver gender.

Predictors of engagement were estimated using Poisson generalized linear mixed models with cluster-level random intercepts. Models were fit to multiply imputed datasets to address missing baseline data, and estimates were pooled across imputations [36]. The primary outcome (number of modules completed) was modeled using an offset for exposure, defined as the number of modules available to each participant. Secondary outcomes (first nonstart and first noncompletion) were modeled as event indicators (1=event, 0=none), with an offset for the number of modules at risk. For participants who experienced an event, the number of modules at risk was defined by the module at which the first disengagement event occurred; for those who did not experience an event, it was defined as the total number of modules available.

Each predictor was tested in a separate model, as the aim was to identify caregiver characteristics associated with engagement that could inform future intervention design and optimization. Predictors were group-mean centered within clusters to isolate within-cluster effects. All models adjusted for the experimental components (dummy-coded for guidance, app design, and digital support). Caregiver age and gender (grand-mean centered) were also included as covariates, except when either variable was the predictor of interest. The potential moderating effect of caregiver gender was examined by including an interaction term between the predictor of interest and gender. Gender was represented both as a group-mean centered variable and as a cluster-level mean to disaggregate individual- and cluster-level effects. Model estimates are reported as incidence rate ratios with 95% CIs and corresponding *P* values. The Benjamini-Hochberg procedure was applied to control the false discovery rate across models, with significance determined using the adjusted critical *P* value thresholds [37].

### Ethical Considerations

Ethics approval was granted by the University of Oxford’s Departmental Research Ethics Committee (R69744/RE001) and the Tanzanian NIMR (NIMR/HQ/R.8s/Vol.IX/3856). Written informed consent was obtained from all study participants at the onboarding session. To support WhatsApp participation and app-server data synchronization, all participants received 1 GB of mobile data per month for the study duration. Participants received US $2 (US $1=TZS 2319.79 as of October 24, 2022) after completing the onboarding session.

## Results

### Characteristics of the Sample

Baseline characteristics for the full sample and by caregiver gender are presented in Table 1. The trial included 614 caregivers (409 women and 205 men), with ages ranging from 18 to 75 years (mean age 35.94, SD 11.84). Independent-samples 2-tailed *t* tests indicated no significant gender differences in age or positive parenting practices. However, women reported significantly higher levels of several socioeconomic and psychosocial stressors compared to men, including financial stress (mean 4.55, SD 2.73 vs mean 3.72, SD 2.73; *P*<.001; Cohen *d*=0.30), food insecurity (mean 7.88, SD 8.09 vs mean 6.17, SD 7.01; *P*=.009; Cohen *d*=0.22), harsh parenting (mean 7.40, SD 6.42 vs mean 5.07, SD 6.14; *P*<.001; Cohen *d*=0.37), and depressive symptoms (mean 8.53, SD 4.12 vs mean 7.80, SD 3.56; *P*=.02; Cohen *d*=0.19). Although effect sizes were small, the findings indicate consistent gender differences in exposure to adversity in the sample.

**Table 1.** Caregiver characteristics overall and by gender, with independent-samples 2-tailed *t* test comparisons.

Measure	Overall		Women		Men		Gender comparison^a^	
	n	Mean (SD)	n	Mean (SD)	n	Mean (SD)	Cohen *d*	*P* value
Age (y)	577	35.94 (11.84)	386	35.70 (11.15)	191	36.43 (13.13)	0.06	.51
Financial stress	574	4.27 (2.75)	379	4.55 (2.73)	195	3.72 (2.73)	0.30	.001
Food insecurity	576	7.30 (7.78)	381	7.88 (8.09)	195	6.17 (7.01)	0.22	.009
Positive parenting	614	10.89 (6.78)	409	10.95 (6.84)	205	10.79 (6.69)	0.02	.78
Harsh parenting	614	6.62 (6.42)	409	7.40 (6.42)	205	5.07 (6.14)	0.37	<.001
Parental depression	614	8.29 (3.95)	409	8.53 (4.12)	205	7.80 (3.56)	0.19	.02

a Gender comparisons were assessed using 2-tailed Welch independent-samples *t *tests. Cohen *d* represents the standardized mean difference between women and men, with positive values indicating higher scores for women. Effect sizes are interpreted as small (*d*=0.20), medium (*d*=0.50), and large (*d*=0.80).

### Program Engagement

#### Descriptives

Of the 614 enrolled caregivers, 97.1% (n=596) initiated the first module, and 90.2% (n=554) completed it. This initial uptake was followed by a marked decline in engagement. By module 2, only 59.8% (n=367) had started the module, and 45.8% (n=281) had completed it. On average, participants started 4.57 (SD 4.13) modules and completed 3.40 (SD 3.64) of the 12 available modules (Table 2). The mean content completion rate across all 12 modules was 35.57% (SD 33.10%) of the program. Completion rates were higher among participants assigned to the guided delivery, unstructured design, and enhanced digital support conditions.

**Table 2.** Modules started and completed: overall and by experimental component^a^.

Variable	Overall	Guidance		App design		Digital support	
		Self-guided (n=330)	Guided (n=284)	Structured (n=332)	Unstructured (n=282)	Basic (n=295)	Enhanced (n=319)
Modules started	4.57 (4.13)	4.22 (3.92)	4.99 (4.33)	4.34 (4.03)	4.84 (4.23)	4.43 (4.04)	4.71 (4.21)
Modules completed	3.40 (3.64)	3.09 (3.41)	3.77 (3.85)	2.91 (3.04)	3.99 (4.16)	3.26 (3.51)	3.54 (3.75)

a Values are presented as mean (SD); possible range, 0-12 modules.

**Figure 1.**
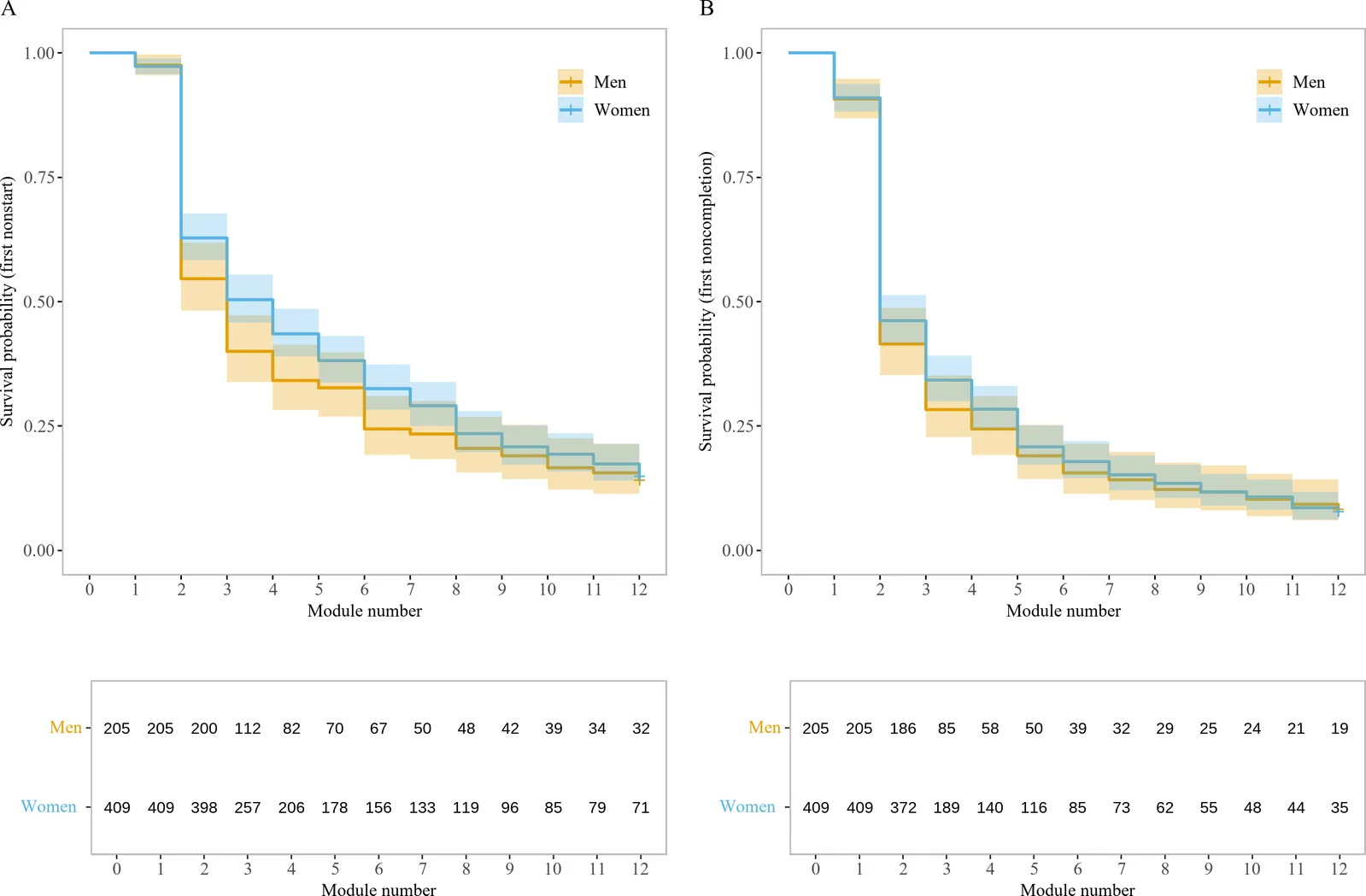
Kaplan-Meier survival curves for (A) first module nonstart and (B) first module noncompletion, stratified by caregiver gender. Curves show the estimated probability of engaging without a first nonstart or noncompletion, respectively, across the 12-module intervention. Risk tables show the number of participants remaining engaged for each module. Shaded areas indicate 95% CIs.

Kaplan-Meier survival analyses indicated that the median retention time among content users was 31 days (95% CI 25‐36), defined as the time between first app launch and final data synchronization with the server. As shown in Figure 1, the risk of disengagement was highest immediately after the first module. Women showed a slightly lower risk of disengagement compared to men for both time to first nonstart and time to first noncompletion, although neither difference was statistically significant.

#### Predictors of Engagement

Each of the 3 engagement outcomes was regressed on 7 baseline predictors, yielding 21 primary hypothesis tests. To control for the false discovery rate within each outcome, the Benjamini-Hochberg procedure was applied separately to each set of 7 tests [37]. The procedure compares each *P* value to a ranked critical value, and only those below the corresponding threshold were considered statistically significant. Statistically significant effects are highlighted in bold in Table 3.

Caregiver gender was the only baseline characteristic significantly associated with module completion. On average, women completed approximately 15% more modules than men (incidence rate ratio 1.15, 95% CI 1.05‐1.27, *P*=.003; Table 3). Caregiver gender showed a similar pattern of association with the two secondary outcomes, with women having lower first nonstart and first noncompletion rates than men, although neither association was statistically significant.

**Table 3.** Baseline predictors of program engagement^a^.

Predictor	Modules completed		First nonstart		First noncomplete	
	IRR^b^ (95% CI)	*P* value	IRR (95% CI)	*P* value	IRR (95% CI)	*P* value
Caregiver gender^c^	1.15 (1.05‐1.27)	.003	0.90 (0.74‐1.08)	.25	0.92 (0.77‐1.10)	.37
Caregiver age	1.02 (0.98‐1.07)	.29	0.96 (0.90‐1.04)	.33	0.99 (0.92‐1.07)	.80
Financial stress	1.02 (1.00‐1.04)	.06	0.98 (0.93‐1.01)	.16	0.98 (0.95‐1.01)	.31
Food insecurity	1.00 (1.00‐1.01)	.67	1.00 (0.99‐1.02)	.52	1.00 (0.98‐1.02)	.41
Positive parenting	1.00 (0.99‐1.02)	.57	1.00 (0.99‐1.02)	.71	1.00 (0.99‐1.02)	.71
Harsh parenting	1.00 (0.99‐1.01)	.87	1.00 (0.98‐1.01)	.70	1.00 (0.99‐1.04)	.93
Parental depression	0.99 (0.98‐1.01)	.22	1.00 (0.98‐1.03)	.83	1.01 (0.99‐1.04)	.32

a All predictors were group-mean centered within clusters. Caregiver gender was dummy-coded (0=men, 1=women), with estimates representing effects for women relative to men (reference group). All models adjusted for the experimental components (dummy-coded guidance, app design, and digital support). Caregiver age and gender (grand-mean centered) were included as covariates unless either was the predictor of interest.

b IRR: incidence rate ratio.

c Effect significant below the Benjamini-Hochberg procedure’s critical value.

#### Moderating Effects of Caregiver Gender

To examine whether the associations between baseline characteristics and engagement outcomes differed by caregiver gender, moderation models were estimated for each of the 7 predictors across all 3 outcomes (Table 4). As with the main-effects models, the Benjamini-Hochberg procedure was applied separately within each outcome to control the false discovery rate. For module completion, interactions between gender and food insecurity and between gender and depressive symptoms approached statistical significance; however, neither met the Benjamini-Hochberg threshold. No significant gender interactions were observed for the secondary outcomes.

**Table 4.** Moderating effects of gender on associations between baseline predictors and program engagement^a^.

Predictor and outcome	Main effect		Interaction effect	
	IRR^b^ (95% CI)	*P* value	IRR (95% CI)	*P* value
Caregiver age				
Modules completed	1.02 (0.98‐1.06)	.41	0.92 (0.84-1.00)	.05
First nonstart	0.97 (0.90‐1.05)	.49	1.09 (0.92‐1.28)	.33
First noncomplete	0.99 (0.93‐1.08)	.95	1.08 (0.92‐1.27)	.33
Financial stress				
Modules completed	1.02 (1.00‐1.04)	.06	0.99 (0.96‐1.03)	.76
First nonstart	0.98 (0.95‐1.01)	.15	1.01 (0.94‐1.09)	.76
First noncomplete	0.98 (0.95‐1.02)	.31	1.00 (0.93‐1.07)	.93
Food insecurity				
Modules completed	1.00 (1.00‐1.01)	.47	0.98 (0.97‐1.00)	.03
First nonstart	1.00 (0.99‐1.02)	.54	1.01 (0.98‐1.04)	.53
First noncomplete	1.00 (0.98‐1.01)	.38	1.02 (0.99‐1.04)	.25
Positive parenting				
Modules completed	1.00 (0.99‐1.01)	.49	1.01 (0.99‐1.03)	.37
First nonstart	1.00 (0.99‐1.02)	.70	0.99 (0.96‐1.03)	.69
First noncomplete	1.00 (0.99‐1.02)	.68	0.99 (0.96‐1.02)	.49
Harsh parenting				
Modules completed	1.00 (0.99‐1.01)	.95	1.01 (1.00‐1.03)	.15
First nonstart	1.00 (0.98‐1.01)	.68	0.99 (0.96‐1.03)	.74
First noncomplete	1.00 (0.99‐1.01)	.86	0.98 (0.95‐1.02)	.33
Caregiver depression				
Modules completed	0.99 (0.98‐1.01)	.38	0.97 (0.94‐1.00)	.04
First nonstart	1.00 (0.98‐1.03)	.86	1.03 (0.97‐1.09)	.38
First noncomplete	1.01 (0.99‐1.04)	.32	1.02 (0.97‐1.08)	.45

a All predictors were group-mean centered within clusters. Caregiver gender was dummy-coded (0=men, 1=women), with interaction terms estimating differential effects for women relative to men (reference group). Gender was represented as both a group-mean centered variable (within-cluster effect) and a cluster-level mean (between-cluster effect). Caregiver age (grand-mean centered) was included as a covariate in all models except when it was the predictor of interest. All models also adjusted for the 3 dummy-coded experimental components (guidance, app design, and digital support).

b IRR: incidence rate ratio.

## Discussion

### Principal Findings

This study provides the first known analysis of caregiver-level predictors of engagement with a digital parenting program in an LMIC, with the notable strength of including a large proportion of men and fathers (205/614, 33.4%). While nearly all participants initiated the program, engagement dropped substantially after onboarding, with the steepest decline occurring after the first module. This mirrors broader trends in digital health interventions, where attrition is typically concentrated in the early stages of program use [38]. These findings highlight the importance of prioritizing retention strategies early in the intervention.

Of the baseline characteristics examined, caregiver gender was the only statistically significant predictor of engagement. Women completed approximately 15% more modules than men, a finding that contrasts with several digital parenting studies in HICs, where engagement is often similar across genders [17]. One possible explanation is that women’s higher engagement may reflect greater exposure to socioeconomic and psychosocial adversity, including financial stress, food insecurity, depressive symptoms, and harsh disciplinary practices. Women reported significantly higher levels of each of these stressors, which may have increased the perceived relevance of the intervention and, in turn, their motivation to engage.

This interpretation aligns with the Health Belief Model [39], which posits that individuals are more likely to adopt health behaviors when they perceive the risks of inaction as high and the benefits of action as tangible. From this perspective, socioeconomic and psychosocial adversity may have served as a cue to action, outweighing emotional or cognitive barriers that might otherwise prevent engagement [40]. That women sustained engagement despite multiple, overlapping stressors is a notable finding. It suggests that digital parenting interventions may offer a feasible and acceptable modality for supporting high-risk caregivers in LMICs.

In contrast, the lower engagement observed among men may reflect a complex interplay of identity, norms, and structural constraints. In Tanzania and many other LMICs, caregiving is typically viewed as a maternal responsibility, while fatherhood is closely tied to provision, protection, and authority [41-43]. When program content is perceived as incongruent with these gendered expectations or fails to affirm men’s aspirations as fathers, it may appear irrelevant or incompatible with their roles. This is echoed in global fatherhood research showing that interventions which acknowledge and support men’s caregiving identities and align with their aspirations tend to enhance perceived relevance and achieve higher engagement [13,44].

Lower engagement among men may also reflect deeper identity tensions under conditions of economic precarity. Qualitative research from Mwanza, Tanzania describes how many men aspire to be nurturing and involved fathers but face conflicting practical and social pressures from work demands, limited time, and community norms [43]. These experiences not only restrict men’s capacity to participate in caregiving but may also lead to internal dissonance when they are unable to fulfill culturally sanctioned roles of fatherhood, a phenomenon that has been described as *moral injury* in fatherhood research [45,46]. Similar findings from Uganda indicate that even when men express egalitarian views, structural constraints and social stigma around caregiving can limit their involvement in parenting programs [47]. These dynamics highlight the need for interventions that actively affirm men’s caregiving identities and address the structural barriers that limit engagement. While women reported greater socioeconomic adversity in the present study, integrating digital parenting programs with broader economic and social protection initiatives may help reduce the opportunity costs of participation for both men and women [48].

Although ParentApp was intentionally designed to support all caregivers rather than assuming mothers as the primary users, these findings suggest that additional strategies may be needed to improve engagement among men. Future iterations could benefit from further co-design with fathers to better understand how intervention content, tone, imagery, and delivery align with men’s caregiving identities and everyday constraints. Emerging work on digital fatherhood interventions illustrates how user-centered design can be used to develop app-based support tailored to fathers’ mental health, well-being, and parenting needs [49]. However, improving engagement among men may require more than tailoring content or delivery; it may also require gender-transformative approaches that actively challenge inequitable norms and support identity shifts in caregiving. As highlighted in a recent systematic review of fatherhood programs in LMICs, gender-transformative interventions foster critical reflection, promote shared decision-making, and engage with normative change at individual and community levels [48]. Promising examples include in-person parenting programs such as REAL Fathers in Uganda [50], Bandebereho in Rwanda [51], and Parenting for Respectability in Uganda [52], which have achieved shifts in father involvement, reductions in intimate partner violence (IPV), and improvements in household dynamics through facilitated dialogue, peer role-modeling, and local leadership.

Emerging evidence suggests that gender-transformative components can be successfully integrated into digital formats. One example is ParentText, a chatbot-based parenting intervention adapted from the PLH program suite and piloted in South Africa and Jamaica. ParentText incorporates content on gender-equitable behaviors and IPV prevention to support shared caregiving, challenge harmful gender norms, and reduce familial violence [53]. Qualitative findings suggest that men valued the anonymity, accessibility, and nonjudgmental tone of the chatbot, which facilitated engagement with sensitive topics such as relationships and caregiving [54]. A parallel pre-post pilot study reported modest reductions in women’s experience of IPV and improvements in men’s attitudes and gender-equitable behaviors. However, overall engagement with the program and with the IPV-related content in particular was limited, especially among men [55]. Together with the findings of the present study, this emerging evidence suggests that moving beyond one-size-fits-all approaches may be key to improving engagement with digital parenting interventions.

### Limitations

Several limitations should be acknowledged when interpreting these findings. First, engagement was operationalized using digital metrics (eg, module completion), which offer behavioral indicators of app use but do not capture content comprehension or real-world application. This reflects a broader challenge in digital health research, where engagement is often equated with usage alone, without consideration of learning or behavior change outcomes [56]. Moreover, these metrics do not capture users’ subjective experiences, such as interest, attention, or emotional resonance, which may be equally important in understanding meaningful engagement [9]. Future research should therefore incorporate multidimensional measures that capture both the quantity and quality of engagement, alongside users’ subjective experiences.

Second, the range of caregiver characteristics assessed at baseline was limited by the brief, app-embedded format of the survey. Key variables such as perceived digital skills and prior experience with technology were not captured, although these factors have been shown to influence engagement with digital interventions [57,58]. The study also did not measure adolescent-level characteristics, such as child mental health symptoms, which have been positively associated with parental engagement in previous research [56]. Accounting for these variables in future studies may improve the precision of engagement models and support more targeted intervention strategies.

Third, while the overall sample size was relatively large, the study may have been underpowered to detect gender interaction effects, especially given the smaller proportion of men in the sample. Future studies should consider adequately powered subgroup analyses to explore gender-specific patterns of engagement. Furthermore, although the sample was drawn from low-income urban and periurban settings, all participants were required to have regular access to a smartphone. As such, findings may not be generalizable to caregivers in rural areas or those with limited access to digital technology. Broader implementation research is needed to understand how structural barriers such as digital access, network reliability, and device sharing influence engagement, including in rural and more resource-constrained settings.

### Conclusions

This study provides important evidence on caregiver engagement with a digital parenting program in an LMIC. Across demographic, socioeconomic, behavioral, and psychological factors, only caregiver gender significantly predicted engagement, with women completing more modules than men. The absence of significant associations with other baseline characteristics provides encouraging evidence that socioeconomic and psychosocial factors may not pose substantial barriers to engagement with ParentApp. However, high rates of attrition among both men and women immediately after onboarding highlight early disengagement as a critical challenge. Addressing this challenge will be essential if digital parenting programs are to achieve meaningful and lasting benefits for children and families in LMICs.

## Abbreviations

HIC: high-income country
IPV: intimate partner violence
LMIC: low- and middle-income country
MOST: Multiphase Optimization Strategy
NIMR: National Institute for Medical Research
PLH: Parenting for Lifelong Health
